# Prognostic value of LAMP3 and TP53 overexpression in benign and malignant gastrointestinal tissues

**DOI:** 10.18632/oncotarget.2643

**Published:** 2014-10-28

**Authors:** Rongwei Sun, Xudong Wang, Huijun Zhu, Haijun Mei, Wei Wang, Shu Zhang, Jianfei Huang

**Affiliations:** ^1^ Department of Pathology, Nantong University Affiliated Hospital, Nantong, Jiangsu, China; ^2^ Department of General Surgery, Nantong University Affiliated Hospital, Nantong, Jiangsu, China; ^3^ Department of Laboratory Medicine, Nantong University Affiliated Hospital, Nantong, Jiangsu, China

**Keywords:** LAMP3, TP53, Prognosis, Gastrointestinal cancer

## Abstract

Lysosomal associated membrane protein 3 (LAMP3) is a newly identified tumor-specific protein. It is a downstream target gene of tumor suppressor TP53 and its expression has been associated with hypoxia-induced metastasis and poor overall survival in cervical and breast cancers. However, little is known of LAMP3 protein expression in gastrointestinal cancer and its prognostic value. We determined protein expression of LAMP3 and TP53 in both gastric (n=750) and colorectal (n=479) tissues by immunohistochemistry analysis on tissue microarray (TMA), their expression was correlated with patients' clinical parameters. LAMP3 and TP53 protein expression was significantly higher in cancerous tissues compared to normal and benign tissues. In both gastric and colorectal cancers, high LAMP3 protein expression (LAMP3+) was significantly associated with tumor stage (P=0.014 and P<0.001). No correlation between LAMP3 and TP53 expression was observed. Patients with high LAMP3 expression but not high TP53 expression had a poor overall survival (for gastric cancer P<0.001, CI: 1.762-4.567; for colorectal cancer P=0.036, CI: 1.062-5.980). Our data suggest that epithelial LAMP3 expression is an independent prognostic marker for gastrointestinal cancer.

## INTRODUCTION

Gastric cancer (GC) is the fourth most common cancer worldwide with an annual incidence of 952,000 and is the second leading cause of global cancer mortality [1]. More than 70% of the GC cases occur in developing countries, with Asia having the highest incidence. Despite the current effort to reduce *Helicobacter Pylori* infection, which is considered the most significant risk factor for GC [2-4], GC remains a prominent public health and an economic burden in Asia [2, 5]. In China, GC is the third leading cause of death from cancer with an age standardized incidence rate of 22.7 per 100,000 [6-7](Cancer Incidence and Mortality Worldwide in 2012, International Agency for Research on Cancer).

Colorectal cancer (CRC) is the third most common cancer in males and the second most common cancer in females worldwide [8], with an annual incidence of 1.4 million. Although the majority of CRC cases occur in developed countries and historically Asia has the lowest incidence, the CRC incidence rate in China has dramatically increased in recent years, with an age standardized incidence rate of 14.2 per 100,000, and ranked the ﬁfth leading cause of death from cancer [9-10].

To improve clinical outcome of gastrointestinal cancer patients, novel molecular prognostic markers are needed as well as improved understanding of the mechanism of tumorigenesis. Lysosomal-associated membrane protein 3 (LAMP3) belongs to the LAMP family proteins. LAMP proteins are highly glycosylated type 1 integral membrane proteins, mainly residing in lysosomal membranes. LAMP3 was originally identified as a marker of mature dendritic cells (CD208, DC-LAMP) [11] as well as a lung specific gene (TSC403) [12]. It is overexpressed in several types of human cancers [12]. Although the precise function of LAMP3 is unknown, recent in vitro and in vivo studies suggest that LAMP3 may be important for tumor metastasis and resistance to therapy: LAMP3 protein was overexpressed in cancer cells from many organs, including cervix, breast, ovary, colon and liver [12]; LAMP3 induces migration and invasion of tumor cells in vitro [13-14]; LAMP3 expression has been associated with resistance to chemotherapy and radiotherapy [15-17]; finally, LAMP3 expression has been associated with lymph node metastasis and poor overall survival [18-20].

TP53 is one of the most important tumor suppressor genes, mutated in over 50% of human malignancies [21]. It regulates DNA repair, cell cycle and apoptosis and therefore plays an essential role in maintaining genetic stability [22]. Because wild type TP53 protein has a short half-life while mutant TP53 proteins are stabilized and show dominant-negative function, TP53 protein detected by immunohistochemistry assay has been widely used as a surrogate marker for TP53 mutation [23-24]. In gastrointestinal cancers, TP53 is one of the most prevalent genetic alterations [25-26], and TP53 protein detection by immunohistochemistry has been associated with better response to chemotherapy [27].

Little is known about the role of LAMP3 in gastrointestinal cancer. Thus far, only two studies have investigated the potential role of LAMP3 in gastrointestinal tumors: Ishigami et al found that the presence of intratumoral LAMP3+ (CD208+) mature interdigitating dendritic cells (IDCs) was inversely correlated with patients' postoperative outcome in GC [28]; Adamsen et al identified LAMP3 as a novel TP53 downstream target gene in colon cancer cells [29]. In the present study, we analyzed epithelial LAMP3 and TP53 expression by immunohistochemistry analysis in both benign and malignant gastric and colorectal tissues using tissue microarrays (TMAs). We correlated epithelial LAMP3 and TP53 expression with clinicopathological characteristics as well as overall survival in patients with gastrointestinal cancers.

## RESULTS

### LAMP3 or TP53 expression in gastrointestinal tissues

LAMP3 protein expression was mainly detected in tumor epithelial cells, only in rare occasions (<5 cases) it was also detected in tumor infiltrating lymphocyte. LAMP3 protein was localized in the cytoplasm while TP53 protein was localized in the nuclei. Using the X-tile software program for TMA data analysis (http://www.tissuearray.org/rimmlab), we first identified significant cutoff point in terms of overall survival in gastric and colorectal cancers. For LAMP3, the cutoff 120 was selected for both gastric and colorectal cancers: score 0-120 was considered low expression while 121-300 was considered high expression. For TP53, the cutoff point 150 was selected for gastrointestinal cancer. For all subsequent analyses, LAMP3 and TP53 protein expression levels were considered either as “Low” or “High” using these cutoff values.

In both gastric and colorectal cancer, more than 50% of cancerous tissues had high LAMP3 expression (LAMP3+), significantly higher than normal surgical margin tissues as well benign tissues (Table 1). Similarly, the frequency of high TP53 expression (TP53+) was significantly higher in cancers than normal surgical margins and benign lesions. Interestingly, high LAMP3 and high TP53 expression (LAMP3+/TP53+) was almost exclusively present in cancerous tissues, only one case each in benign gastric and colorectal benign tissues had LAMP3+/TP53+ staining.

**Tabcle 1:** LAMP3 and TP53 expression in gastrointestinal benign and malignant tissues. LAMP3+ represents high LAMP3 expression, TP53+ represents high TP53 expression, and LAMP3+/TP53+ represents high LAMP3 and high TP53 expression

Characteristic	n	LAMP3+	Pearson ϰ^2^	*P*	P53+	Pearson ϰ^2^	*P*	LAMP3+/P53+	Pearson ϰ^2^	*P*
Stomach			60.895	<0.001*		59.461	<0.001*		37.433	<0.001*
Chronic gastritis	23	8(34.78)			0(0.00)			0(0.00)		
Intestinal metaplasia	18	8(44.44)			0(0.00)			0(00.00)		
Low-grade intraepithelial neoplasia	32	12(37.50)			3(9.38)			1(3.13)		
High-grade intraepithelial neoplasia	22	11(50.00)			1(4.55)			0(0.00)		
Cancer	528	290(54.92)			147(27.84)			84(15.91)		
Surgical margin	127	22(17.32)			3(2.36)			0(0.00)		
Colon and Rectum			83.171	<0.001*		66.146	<0.001*		44.246	<0.001*
Chronic colitis	23	2(8.70)			1(4.35)			0(0.00)		
Low-grade intraepithelial neoplasia	41	6(14.63)			1(2.44)			0(0.00)		
High-grade intraepithelial neoplasia	21	8(38.10)			2(9.52)			1(4.76)		
Cancer	197	115(58.38)			49(24.87)			31(15.74)		
Surgical margin	194	37(19.07)			0(0.00)			0(0.00)		

* *p*<0.05;

### Association of LAMP3 and TP53 expression with clinicopathologic characteristics in gastrointestinal cancers

Next, we examined the correlation between LAMP3 or TP53 protein expression and clinical parameters among gastrointestinal cancer patients.

In gastric cancer, high LAMP3 expression was significantly associated with tumor stage (P=0.014), especially with lymph node metastasis (P=0.003); while high TP53 expression was significantly associated with patient age (P=0.006), tumor size (P=0.03), preoperative CEA (P=0.013) and CA19-9 (P=0.032) levels (Table 2). High LAMP3 and high TP53 expression (LAMP3+/TP53+) was significantly associated with patient age (P=0.007), tumor stage (P=0.026), and preoperative CEA level (P=0.013), marginally associated with lymph node metastasis (P=0.057). No correlation between LAMP3 and TP53 expression was detected.

**Table 2:** Association of high expression of LAMP3 and TP53 with clinicopathological characteristics in gastric cancer patients

Characteristic	n	LAMP3+	earson ϰ^2^	*P*	P53+	Pearson ϰ^2^	*P*	LAMP3+/P53+	Pearson ϰ^2^	*P*
Total	528	290(54.92)			147(27.84)			84(15.91)		
Gender			0.108	0.742		0.082	0.774		0.001	0.976
Male	389	212(54.50)			107(27.51)			62(15.94)		
Female	139	78(56.12)			40(28.78)			22(15.83)		
Age			0.577	0.448		7.639	0.006*		7.294	0.007*
<60	208	110(52.88)			44(21.15)			22(10.58)		
≥60	320	180(56.25)			103(32.19)			62(19.38)		
Histological type			8.013	0.091		3.363	0.499		5.324	0.256
Tubular	444	247(55.63)			128(28.83)			77(17.34)		
Mixed (Tubular and mucinous)	18	14(77.78)			2(11.11)			2(11.11)		
Mucinous	27	13(48.15)			8(29.63)			3(11.11)		
signet ring cell	24	11(45.83)			5(20.83)			2(8.33)		
Others^a^	15	5(33.33)			4(26.67)			0(0.00)		
Differentiation			0.182	0.913		1.584	0.453		2.928	0.231
Well	21	12(57.14)			6(28.57)			4(19.05)		
Middle	136	76(55.88)			45(33.09)			29(21.32)		
Poor	298	161(54.03)			81(27.18)			44(14.77)		
Others^b^	73	41			15			7		
TNM stage			17.537	0.014*		11.937	0.103		15.908	0.026*
0	19	8(42.11)			3(15.79)			1(5.26)		
Ia	33	12(36.36)			8(24.24)			2(6.06)		
Ib	60	30(50.00)			15(25.00)			8(13.33)		
IIa	112	55(49.11)			25(22.32)			12(10.71)		
IIb	70	38(54.29)			18(25.71)			10(14.29)		
IIIa	92	52(56.52)			33(35.87)			24(26.09)		
IIIb	94	66(70.21)			35(37.23)			20(21.28)		
IIIc+IV	48	29(60.42)			10(20.83)			7(14.58)		
T			7.192	0.126		10.741	0.030*		7.689	0.104
Tis	19	8(42.11)			3(15.79)			1(5.26)		
T1	50	22(44.00)			12(24.00)			5(10.00)		
T2	104	59(56.73)			18(17.31)			11(10.58)		
T3	313	172(54.95)			101(37.27)			58(18.53)		
T4	42	39(69.05)			13(30.95)			9(21.43)		
N			14.141	0.003*		2.172	0.537		7.512	0.057
N0	202	92(45.54)			50(24.75)			22(10.89)		
N1	97	54(55.67)			26(26.80)			16(16.49)		
N2	108	64(9.26)			33(30.56)			24(22.22)		
N3	121	80(66.12)			38(31.40)			22(18.18)		
M			0.842	0.359		1.577	0.209		0.171	0.680
M0	491	267(54.38)			140(28.51)			79(16.09)		
M1	37	23(62.16)			7(18.92)			5(13.51)		
Preoperative CEA, ng/ml			0.355	0.551		6.218	0.013*		6.173	0.013*
≤ 5	226	117(51.77)			52(23.01)			26(11.50)		
> 5	68	38(55.88)			26(38.24)			16(25.53)		
Unknown	234	135			69			42		
Preoperative CA199, U/ml			0.001	0.977		4.603	0.032*		2.665	0.103
≤ 37	236	125(52.97)			55(23.31)			29(12.29)		
> 37	47	25(53.19)			18(38.30)			10(21.28)		
Unknown	245	140			74			45		
P53			0.405	0.525		-	-		-	-
High	147	84(57.14)			-			-		
Low	381	206(54.07)			-			-		
LAMP3			-	-		0.405	0.525		-	-
High	290	-			84(28.97)			-		
Low	238	-			63(26.47)			-		

* *p*<0.05;

a others: Papillary adenocarcinoma, 4 cases; Adeno-squamous carcinoma, 3 cases; Squamous cell carcinoma, 4 cases; Undifferentiated carcinoma, 2 cases; Neuroendocrine carcinoma, 2 cases

b others: besides Tubular and Papillary adenocarcinoma.

In colorectal cancer, high LAMP3 expression was significantly associated with tumor stage (p<0.001), especially with tumor size (P<0.001), and preoperative CEA level (P=0.016); while high TP53 expression was marginally associated with tumor histological type (P=0.069) (Table 3). High LAMP3 and high TP53 (LAMP3+/TP53+) expression was marginally associated with tumor size (P=0.072). No correlation between LAMP3 and TP53 expression was detected.

**Table 3:** Association of high expression of LAMP3 and TP53 with clinicopathological characteristics in colorectal cancer patients

Characteristic	n	LAMP3+	Pearson ϰ^2^	*P*	P53+	Pearson ϰ^2^	*P*	LAMP3+/P53+	Pearson ϰ^2^	*P*
Total	197	115(58.38)			49(24.87)			31(15.74)		
Gender			0.068	0.794		0.795	0.373		0.162	0.687
Male	127	75(59.06)			29(22.83)			19(14.96)		
Female	70	40(57.14)			20(28.57)			12(17.14)		
Age			0.012	0.911		1.055	0.304		0.200	0.655
<60	64	37(64.06)			13(20.31)			9(14.06)		
≥60	133	78(64.66)			36(27.07)			22(16.54)		
Location			2.040	0.153		0.122	0.727		0.007	0.935
Colon	145	89(61.38)			37(25.52)			23(15.86)		
Rectum	52	26(50.00)			12(23.08)			8(15.38)		
Histological type			0.282	0.595		3.301	0.069		0.825	0.364
Tubular	175	101(57.71)			47(26.86)			29(16.57)		
Other^a^	22	14(63.64)			2(9.09)			2(9.09)		
Differentiation			0.239	0.625		0.230	0.632		0.756	0.385
Poor	159	93(58.49)			42(26.42)			20(16.67)		
Well and middle	19	10(52.63)			6(31.58)			6(24.00)		
Other^b^	19	12			1			1		
TNM stage			16.044	<0.001*		1.785	0.410		3.133	0.209
0-I	45	17(37.78)			8(17.78)			4(8.89)		
II	77	57(74.03)			22(28.57)			16(20.78)		
III +IV	75	41(54.67)			19(25.33)			11(14.67)		
T			12.634	<0.001*		1.923	0.166		3.233	0.072
Tis+ T1+T2	51	19(37.25)			9(17.65)			4(7.84)		
T3, 4b	146	96(65.75)			40(27.40)			27(18.49)		
N			0.899	0.826		1.115	0.773		4.010	0.260
N0	124	75(60.48)			30(24.19)			20(16.13)		
N1a	38	21(55.26)			9(23.68)			5(13.16)		
N1b	18	9(50.00)			4(22.22)			1(5.65)		
N2a,b	17	10(58.82)			6(35.29)			5(29.41)		
M			0.988	0.320		0.823	0.364		1.169	0.280
M0	186	107(57.53)			45(24.19)			28(15.05)		
M1a+1b	11	8(72.73)			4(36.36)			3(27.27)		
Preoperative CEA, ng/ml			5.801	0.016*		0.242	0.623		0.939	0.333
≤ 5	120	63(52.50)			34(28.33)			20(16.67)		
> 5	24	19(79.17)			8(33.33)			6(25.00)		
Unknown	53	33			7			5	-	-
P53			0.642	0.423		-	-			
High	49	31(63.27)			-			-		
Low	148	84(56.76)			-			-		
Lamp3			-	-		0.642	0.423		-	-
High	115	-			31(26.96)			-		
Low	82	-			18(21.95)			-		

a others: Mixed (Tubular and mucinous) adenocarcinoma, 10 cases; Mucinous carcinoma, 6 cases; signet ring cell carcinoma, 2 cases; Adeno-squamous carcinoma, 1 cases; Papillary adenocarcinoma, 3 cases.

b others: besides Tubular and Papillary adenocarcinoma.

* *p*<0.05

### Prognostic value of LAMP3 and TP53 protein expression in gastrointestinal cancer

We also determined prognostic factors in gastrointestinal cancers using both univariate and multivariate analysis.

In gastric cancer, high LAMP3 expression (HR, 2.766, 95% CI, 2.138-3.578; P<0.001) was significantly associated with poor overall survival in univariate analysis, along with previously reported prognostic markers, including age (HR, 1.365, 95% CI, 1.069-1.742; P=0.012), differentiation (HR, 1.620, 95% CI, 1.269-2.068; P<0.001), tumor stage (HR, 1.605, 95% CI, 1.493-1.726; P<0.001), preoperative CEA (HR, 2.325, 95% CI, 1.659-3.257; P<0.001) and CA19-9 (HR, 2.693, 95% CI, 1.865-3.889; P<0.001) levels. High TP53 expression was significantly associated with poor overall survival (HR, 1.420, 95% CI, 1.111-1.814; P=0.005), and high LAMP3 and high TP53 (LAMP3+/TP53+) was significantly associated with poor overall survival (HR, 1.960, 95% CI, 1.488-2.581; P<0.001). In multivariate analysis, only high LAMP3 expression remained significantly associated with poor overall survival (HR, 2.836, 95% CI, 1.762-4.567; P<0.001), so did tumor stage (HR, 1.641, 95% CI, 1.460-1.844; P<0.001), preoperative CEA (HR, 2.176, 95% CI, 1.433-3.304; P<0.001) and CA19-9 levels (HR, 1.852, 95% CI, 1.170-2.931; P=0.009) (Table 4).

**Table 4:** Univariate and multivariate analysis of prognostic markers for overall survival in gastric cancer

	Univariate analysis				Multivariate analysis			
	HR	P >|z|	95% CI		HR	P >|z|	95% CI	
LAMP3 expression	2.766	<0.001*	2.138	3.578	2.836	<0.001*	1.762	4.567
High vs Low								
P53 expression	1.420	0.005*	1.111	1.814	1.510	0.230	0.770	2.959
High vs Low								
LAMP3+P53+	1.960	<0.001*	1.488	2.581	0.543	0.145	0.239	1.234
Lamp3+P53+ vs Non-Lamp3+P53+								
Age (years)	1.365	0.012*	1.069	1.742	0.779	0.182	0.539	1.124
≤60 vs >60								
Gender	0.924	0.555	0.712	1.200				
Male vs Female								
Histological type	0.967	0.586	0.856	1.092				
Tubular vs Mixed (Tubular and mucinous) vs Mucinous vs Signet ring cell carcinoma vs others^a^								
Differentiation	1.620	<0.001*	1.269	2.068	1.3412	0.071	0.971	2.053
Well vs Middle vs Poor								
TNM stage	1.605	<0.001*	1.493	1.726	1.641	<0.001*	1.460	1.844
0 vs Ia vs Ib vs IIa vs IIb vs IIIa vs IIIb vs IIIc and IV								
T	2.138	<0.001*	1.807	2.529				
Tis vs T1 vs T2 vs T3 vs T4								
N	1.760	<0.001*	1.591	1.948				
N0 vs N1 vs N2 vs N3								
M	2.968	<0.001*	2.040	4.320				
M0 vs M1								
Preoperative CEA, ng/ml	2.325	<0.001*	1.659	3.257	2.176	<0.001*	1.433	3.304
≤5 vs >5								
Preoperative CA199, U/ml	2.693	<0.001*	1.865	3.889	1.852	0.009*	1.170	2.931
≤37 vs >37								

* *p*<0.05;

a others: Papillary adenocarcinoma, 4 cases; Adeno-squamous carcinoma, 3 cases; Squamous cell carcinoma, 4 cases; Undifferentiated carcinoma, 2 cases; Neuroendocrine carcinoma, 2 cases

In colorectal cancer, high LAMP3 expression was significantly associated with poor overall survival in univariate analysis (HR, 2.919, 95% CI, 1.666-5.114; P<0.001), along with previously reported prognostic markers, including differentiation (HR, 4.047, 95% CI, 2.227-7.353; P<0.001), tumor stage (HR, 2.126, 95% CI, 1.500-3.012; P=0.003), and preoperative CEA level (HR, 2.510, 95% CI, 1.354-4.651; P=0.003). High TP53 expression was significantly associated with poor overall survival (HR, 2.051, 95% CI, 1.260-3.338; P=0.004), and high LAMP3 and high TP53 (LAMP3+/TP53+) was significantly associated with poor overall survival (HR, 3.304, 95% CI, 1.813-5.076; P<0.001). In multivariate analysis, high LAMP3 expression remained significantly associated with poor overall survival (HR, 2.519, 95% CI, 1.062-5.980; P=0.036), as did tumor differentiation (HR, 4.741, 95% CI, 2.252-9.982; P<0.001) and tumor stage (HR, 1.988, 95% CI, 1.260-3.137; P=0.003) (Table 5).

**Table 5:** Univariate and multivariate analysis of prognostic markers for overall survival in colorectal cancer

	Univariate analysis				Multivariate analysis			
	HR	P >|z|	95% CI		HR	P >|z|	95% CI	
LAMP3 expression	2.919	<0.001*	1.666	5.114	2.519	0.036*	1.062	5.980
High vs Low								
P53 expression	2.051	0.004*	1.260	3.338	0.914	0.884	0.274	3.052
High vs Low								
LAMP3+P53+	3.304	<0.001*	1.813	5.076	1.965	0.340	0.491	7.860
Lamp3+/P53+vs Non-Lamp3+/P53+								
Age (years)	0.976	0.926	0.592	1.611				
≤60 vs >60								
Gender	1.235	0.414	0.744	2.050				
Male vs Female								
Location	1.311	0.295	0.790	2.175				
Colon vs Rectum								
Histological type	1.715	0.245	0.690	4.262				
Tubular and Papillary vs Others^a^								
Differentiation	4.047	<0.001*	2.227	7.353	4.741	<0.001*	2.252	9.982
Well and Middle vs Poor								
TNM stage	2.126	<0.001*	1.500	3.012	1.988	0.003*	1.260	3.137
0 and I vs II vs III and IV								
T	10.021	<0.001*	3.149	31.889				
Tis+T1 +T2 vs T3 and 4a								
N	1.500	<0.001*	1.213	1.856				
N0 vs N1a vs N1b vs N2a and 2b								
M	5.460	<0.001*	2.756	10.8916				
M0 vs M1a+1b								
Preoperative CEA, ng/ml	2.510	0.003*	1.354	4.651	1.425	0.296	0.734	2.767
≤5 vs >5								

* *p*<0.05;

a others: Mixed (Tubular and mucinous) adenocarcinoma, 10 cases; Mucinous carcinoma, 6 cases; signet ring cell carcinoma, 2 cases; Adeno-squamous carcinoma, 1 cases; Papillary adenocarcinoma, 3 cases.

**Figure 1:**
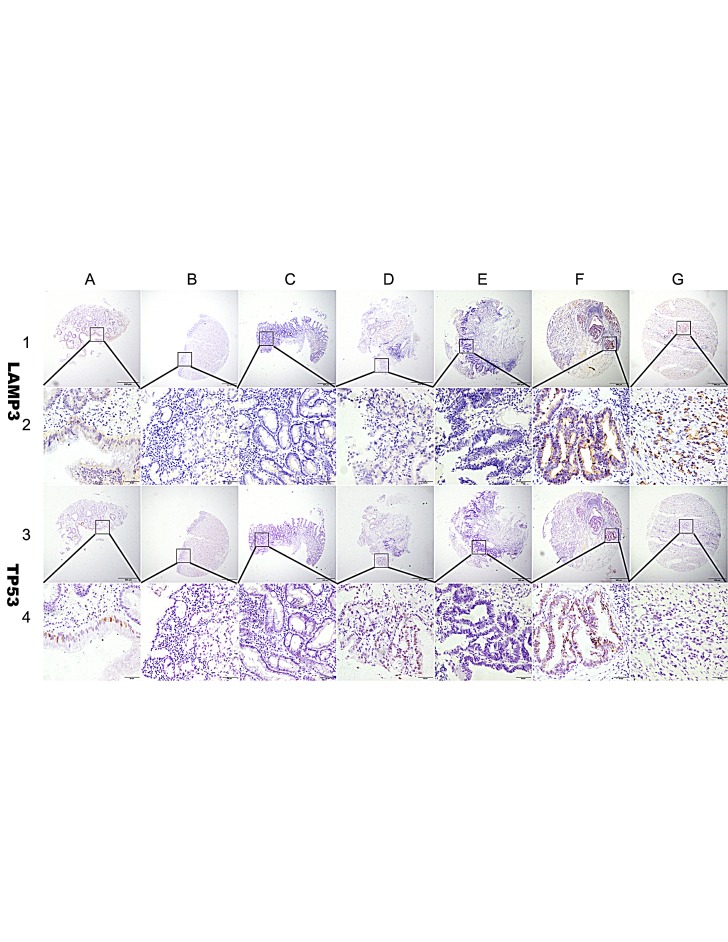
Representation of LAMP3 and TP53 protein expression in gastric benign and malignant tissues on TMA sections Column A: normal surgical margin of gastric cancer with low LAMP3 expression (IHC score, 80) and low TP53 expression (IHC score, 30); column B: chronic gastritis with no LAMP3 expression (IHC score, 0) and negative TP53 expression(IHC score, 0); column C: intestinal metaplasia with low LAMP3 expression (IHC score, 30) and no TP53 expression(IHC score, 0); column D: low-grade intraepithelial neoplasia with low LAMP3 expression (IHC score, 60) and high TP53 expression (IHC score, 180); column E: high-grade intraepithelial neoplasia with low LAMP3 expression (IHC score, 40) and no TP53 expression (IHC score, 0); column F: well differentiated gastric cancer with high LAMP3 expression (IHC score, 200) and high TP53 expression (IHC score, 210); column G: poorly differentiated gastric cancer with high LAMP3 expression (IHC score, 270)and no TP53 expression (IHC score, 0). Row 1 and 2 are LAMP3 staining with x40 (bar=500 μm) and x400 (bar=50 μm) magnification respectively, and row 3 and 4 are TP53 staining with with x40 (bar=500 μm) and x400 (bar=50 μm) magnification respectively.

**Figure 2:**
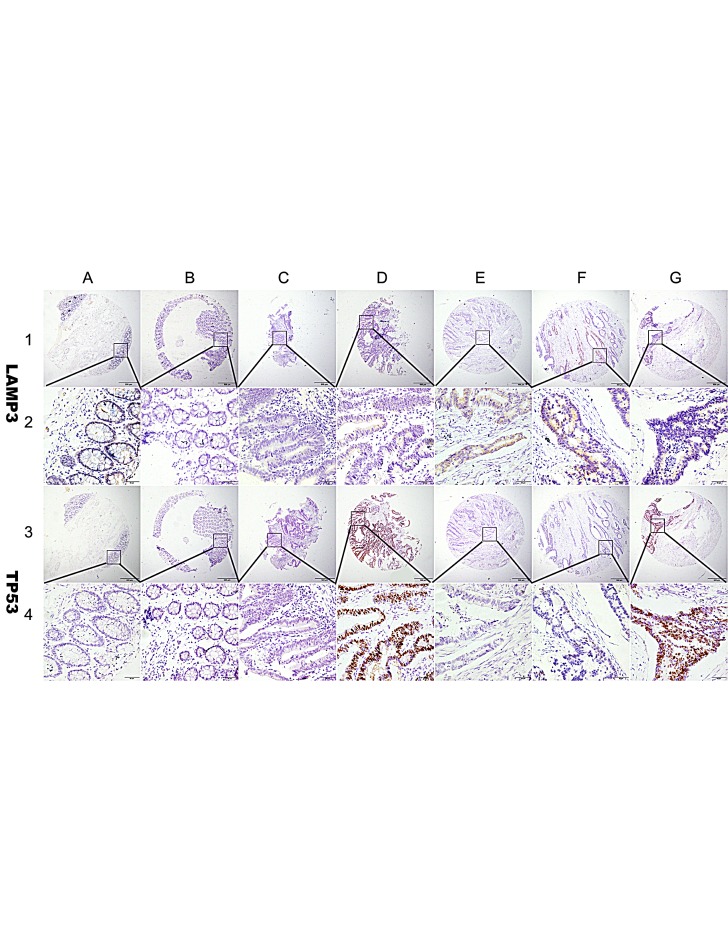
Representation of LAMP3 and TP53 protein expression in colorectal benign and malignant tissues on TMA sections Column A: normal surgical margin of colorectal cancer with low LAMP3 expression (IHC score, 60) and no TP53 expression (IHC score, 0); column B: chronic colitis with no LAMP3 expression (IHC score, 0) and TP53 expression (IHC score, 0); column C: intestinal metaplasia with low LAMP3 expression (IHC score, 40) and TP53 expression (IHC score, 40); column D: low-grade intraepithelial neoplasia with low LAMP3 expression (IHC score, 100) and high TP53 expression (IHC score, 300); column E: high-grade intraepithelial neoplasia with low LAMP3 expression (IHC score, 120) and no P53 expression (IHC score, 0); column F: low-grade colorectal cancer with high LAMP3 expression (IHC score, 180) and no TP53 expression (IHC score, 0); column G: high grade colorectal cancer with low LAMP3 expression (IHC score, 100) and high TP53 expression (IHC score, 270). Row 1 and 2 are LAMP3 staining with x40 (bar=500 μm) and x400 (bar=50 μm) magnification respectively, and row 3 and 4 are TP53 staining with with x40 (bar=500 μm) and x400 (bar=50 μm) magnification respectively.

**Figure 3:**
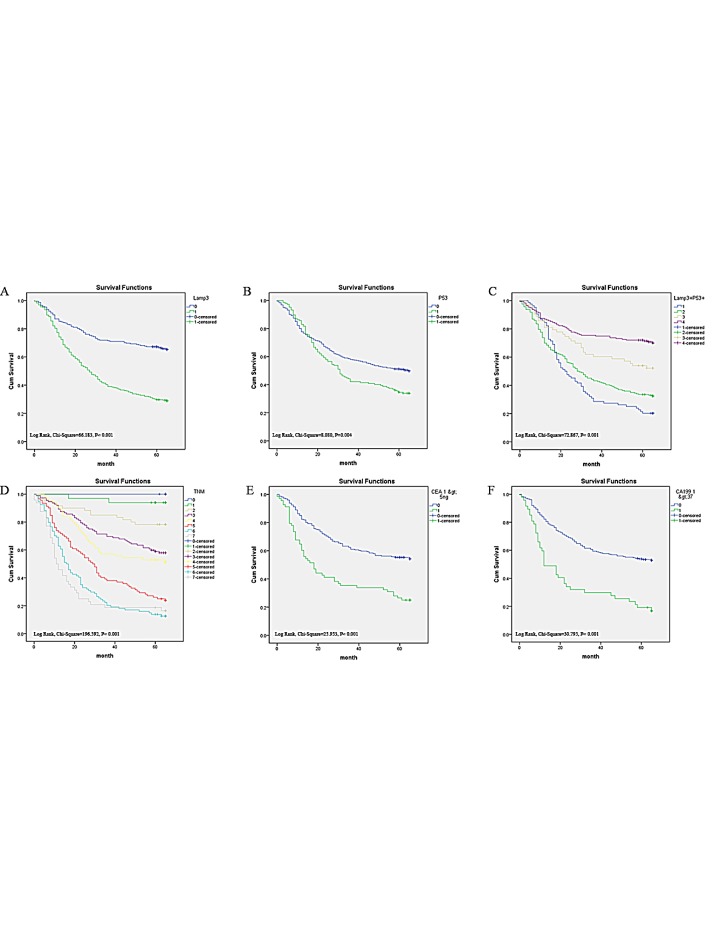
Survival curves of gastric cancer by the Kaplan-Meier method and the log-rank test A: overall survival curves of LAMP3+ (green line, 1) and LAMP3- (blue line, 0); B: overall survival curves of TP53+ (green line, 1) and TP53- (blue line, 0); C: overall survival curves of LAMP3+/TP53+ (blue line, 1), LAMP3+/TP53- (green line, 2), LAMP3-/TP53+ (yellow line, 3), and LAMP3-/TP53- (purple line, 4); D: overall survival curves by stage, TNM IIIc and IV (gray line, 7), TNM IIIb (lightblue line, 6), TNM IIIa (red line, 5), TNM IIb (yellow line, 4), TNM IIa (purple line, 3), TNM Ib (lightyellow line, 2), TNM Ia (green line, 1) and TNM 0 (blue line, 0); E: overall survival curves by preoperative CEA, high (green line, 1) and low (blue line, 0); F: overall survival curves by preoperative CA19-9 level, high (green line, 1) and low (blue line, 0).

**Figure 4:**
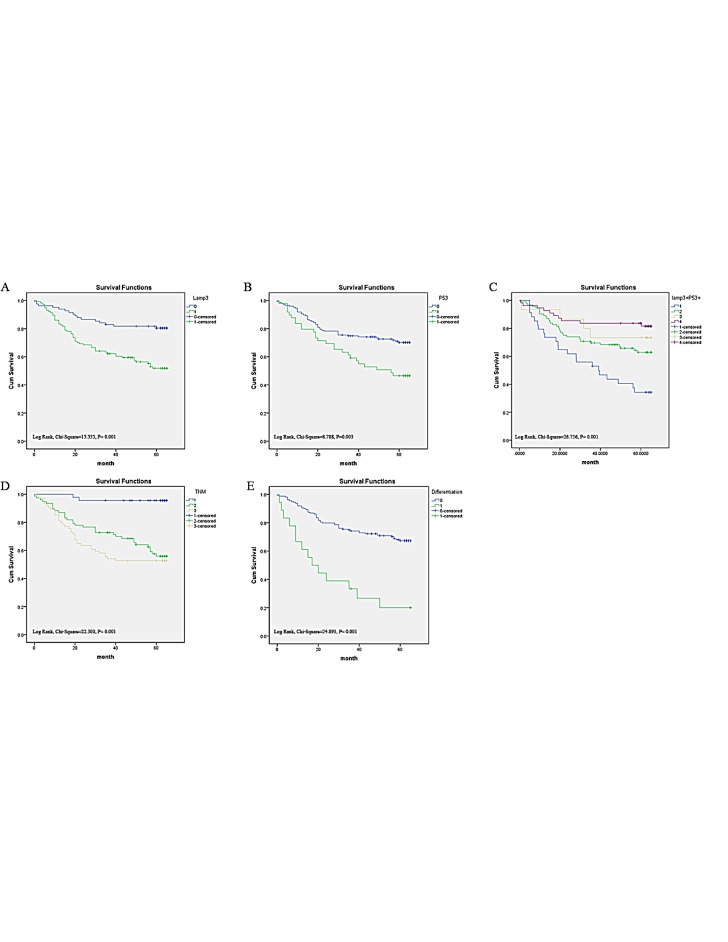
Survival curves of colorectal cancer by the Kaplan-Meier method and the log-rank test A: overall survival curves of LAMP3+ (green line, 1) and LAMP3- (blue line, 0); B: overall survival curves of TP53+ (green line, 1) and TP53- (blue line, 0); C: overall survival curves of LAMP3+/TP53+ (blue line, 1), LAMP3+/TP53- (green line, 2), LAMP3-/TP53+ (yellow line, 3), and LAMP3-/TP53- (purple line, 4); D: overall survival curves by stage, TNM III and IV (yellow line, 3), TNM II (green line, 2), and TNM 1 and 0 (blue line, 1); E: overall survival curves by differentiation, poor differentiation (green line, 1), well and middle differentiation (blue line, 0).

## DISCUSSION

In this study, we have determined LAMP3 and TP53 protein expression in gastrointestinal tissues by immunohistochemistry analysis on tissue microarray (TMA). We found that both LAMP3 and TP53 protein expression were significantly higher in cancerous tissues than in normal and benign tissues. In both gastric and colorectal cancers, we found high LAMP3 protein expression was associated with tumor stage, though we did not detect correlation between LAMP3 and TP53 expression. In both univariate and multivariate analysis, we found high LAMP3 expression was significantly associated with patients' poor overall survival.

To our best knowledge, this is the first study investigating epithelial LAMP3 protein expression as well as potential LAMP3 and TP53 protein interaction in gastrointestinal cancers. LAMP3 was originally characterized as a molecular marker for mature intedigitating dendritic cells (DC) (DC-LAMP) [11]. In fact, Ishigami et al demonstrated that the presence of LAMP3+ tumor infiltrating mature DC cells had prognostic value in gastric cancer, but they did not investigate LAMP3 expression in tumor epithelial cells [28]. In our study, we mainly detected LAMP3 protein expression in epithelial cells, only in rare occasions (<5 cases) did we also observe tumor infiltrating lymphocytes positive for LAMP3 protein. The difference between Ishigami's study and ours might be due to the different antibodies used: monoclonal antibody (Ishigami study) vs polyclonal antibody (our study); or difference in study populations: 47% (Ishigami's study) vs 22% (our study) stage I gastric cancer patients. LAMP3 protein expression has been detected in both DC and epithelial cells in other types of cancer. Liu et al identified LAMP3+ (CD208+) mature DCs in the margin of cancerous tissues of esophageal squamous cell carcinoma [30]. Expression of LAMP3 protein in both dendritic cell and tumor epithelial cell had prognostic value in breast cancer [18, 31]. In cervical cancer, only epithelial LAMP3+ expression was detected [14]. Future studies are needed to resolve discrepancies between DC and tumor epithelial cell LAMP3 expression in cancer tissues.

To investigate downstream TP53 target genes involved in 5-fluorouracil (5-FU) resistance, Adamsen et al compared gene expression changes after 5-FU treatment in colon cancer cells with wild type or mutant TP53 [29]. They identified LAMP3 gene as being upregulated by TP53 gene but downregulated by 5-FU in colon cancer cell lines with mutant TP53. In this clinical study, we observed neither protein expression correlation between LAMP3 and TP53, nor prognostic synergy between LAMP3 and TP53 expression on overall survival: only LAMP3 expression but not the combination of LAMP3 and TP53 was an independent prognostic marker for survival.

Our data indicated that high TP53 expression was associated with poor overall survival in both gastric and colorectal cancer [32-34]. This is consistent with studies in colon and breast cancers, but in contrast with a recent study in gastric cancer where high TP53 expression is associated with better response to chemotherapy [27]. Because not all TP53 mutations lead to accumulation of TP53 protein [35-36], cautions should be taken when interpreting TP53 IHC data. Havrilesky et al demonstrated that TP53 mutation but not TP53 overexpression was associated patients' survival in ovarian cancer [24]. Future studies are needed to directly compare TP53 protein expression with TP53 mutation detected by sequencing in gastrointestinal cancers and their association with overall survival.

LAMP3 is the newest member of the LAMP protein family, which is a lysosomal-associated membrane protein that is rarely expressed in normal cells but abundant in cancer cells. Because LAMP genes are major carriers of sialylated lewis x antigens, it has been hypothesized that LAMP genes are involved in tumor invasion and metastasis by regulating tumor adhesion to endothelial cells through tumor associated sialylated lewis x antigen and E-selectin on endothelial cells [12].

Other studies indicate that LAMP3 is a novel hypoxia-regulated gene and a mediator of hypoxia induced metastasis [37]. Both LAMP3 mRNA and protein are induced by hypoxia. In cervical cancer, expression of LAMP3 is associated with hypoxia and mediates hypoxia-driven nodal metastasis through regulating cell migration [14, 19]. In breast cancer xenografts, LAMP3 protein expression colocalizes with hypoxic areas and is associated with locoregional recurrence[18]. Mechanistically, it has been shown that tumor hypoxia induces unfolded protein response (UPR) pathway, which in turn induces LAMP3 via the PKR-like ER kinase (PERK)/activating transcription factor 4 (ATF4)-arm of the UPR [13, 37].

Our study has several limitations: first, it is a retrospective observational study, the conclusions might not be applicable to the general population. Larger prospective studies are needed to confirm our findings. Secondly, we have used TMA to analyze LAMP3 and TP53 protein level, the expression pattern might not represent the expression pattern of the whole tissue, thus introducing potential biases in the data. Thirdly, IHC data are semiquantitative, additional methods are needed to evaluate and confirm LAMP3 and TP53 expression in tumor cells. Finally, we do not know whether LAMP3 protein is induced by hypoxia in gastrointestinal cancer. Future in vitro studies are needed to investigate the mechanism of LAMP3 in tumorigenesis.

In conclusion, we have shown that high LAMP3 protein expression is an independent prognostic marker in gastrointestinal cancers. Because of the role of LAMP3 in tumor-associated hypoxia, future research is warranted to investigate whether LAMP3 plays a role in hypoxia-associated treatment failure and whether LAMP3 is a valid novel therapy target in gastrointestinal cancers.

## METHODS

### Human tissue specimens and patient clinical information

A total of 1229 formalin-fixed paraffin-embedded (FFPE) tissue samples were collected from 908 patients. These include 761 stomach tissues: 539 cancer, 127 matched normal surgical margins, 23 chronic gastritis, 18 intestinal metaplasia, 32 low-grade intraepithelial neoplasia, and 22 high-grade intraepithelial neoplasia; 479 colon and rectum tissues: 200 cancer, 194 matched normal surgical margins, 23 chronic colitis, 41 low-grade intraepithelial neoplasia, and 21 high-grade intraepithelial neoplasia. All tissue blocks were obtained from the Department of Pathology, Affiliated Hospital of Nantong University from 2003 to 2010. Clinical characteristics of cancer patients were extracted from their medical record, including: age, sex, histological type, differentiation grade, tumor stage, preoperative serum CEA and CA19-9 levels. None of the cancer patients received any types of treatments (radiation therapy, chemotherapy, or immunotherapy) before surgery. Overall survival (OS) was defined as the period from initial biopsy confirmed diagnosis to death. Patients who were alive at the last follow-up date were censored from the analysis. The study protocol was approved by the Human Research Ethics Committee of the Affiliated Hospital of Nantong University, Jiangsu, China.

### Tissue microarray (TMA) construction and immunohistochemistry analysis (IHC)

TMA was generated using the manual Tissue Microarrayer System Quick Ray (UT06, UNITMA, Korea) in the Department of Clinical Pathology, Nantong University Hospital, Jiangsu, China. Specifically, core tissue biopsies (2 mm in diameter) were taken from ~70 individual FFPE blocks and arranged in a new recipient paraffin block. A total 22 TMAs were made, including 13 gastric TMAs and 9 colorectal TMAs. Four-micron sections were cut and placed on super frost-charged glass microscope slides to generate TMA slides.

Tissue sections were deparafﬁnized and rehydrated through graded alcohols. Endogenous peroxidase activity was blocked by incubation in 3% H_2_O_2_. Antigen retrieval was carried out with 0.01 M citrate buffer pH 6.0 and microwave heat induction. LAMP3 was detected by rabbit polyclonal anti-human LAMP3 antibody (dilution 1:100) (Abcam, ab111090), and TP53 was detected by rabbit polyclonal anti-human TP53 antibody (dilution 1:100) (DAKO, M3629). Reactions were detected with Envision+^TM^ peroxidase kit (Dako, Carpinteria, CA, USA). Color development was accomplished by incubating with 3,3′-diaminobenzidine plus (Dako, Carpinteria, CA, USA), counterstained with Hematoxylin, dehydrated through graded alcohols, cleared in xylene, and coverslipped with permanent mounting media.

All cases were reviewed and scored without knowledge of clinical characteristics. The expression of LAMP3 and TP53 was scored using the semi-quantitative H-score method, taking into account both the staining intensity and the percentage of cells at that intensity [38]. The staining intensity was scored as 0 (no staining), 1+ (weak staining), 2+ (moderate staining), or 3+ (intense staining). For each of the four staining intensity scores, the percentage of cells stained at the respective intensity was determined and multiplied by the intensity score to yield an intensity percentage score. The final staining scores were then calculated from the sum of the four intensity percentage scores; thus the staining score had a minimum value of 0 (no staining) and a maximum of 300 (100% of cells with 3+ staining intensity).

### Statistical analysis

For statistical analysis, the continuous LAMP3 and TP53 expression data from IHC were first converted into dichotic data (low vs high) using specific cutoff values, which were selected to be significant in terms of overall survival (OS) using the X-tile software program (The Rimm Lab at Yale University; http://www.tissuearray.org/rimmlab) [20, 39].

Student t test and Pearson χ2 test were used to determine the statistical significance of differences between comparison groups. The correlation between LAMP3 and TP53 protein expression was calculated using Spearman's test. The cumulative patient survival was estimated using the Kaplan-Meier method, and a log-rank test was used to compare the survival curves. A Cox proportional hazards model was used to calculate univariate and multivariate hazard ratios for the variables. A P-value of less than 0.05 was considered statistically significant. All statistical analyses were carried out using the SPSS 20.0 statistical software package (SPSS Inc., Chicago, IL).
